# Identification of Adiabatic Temperature Rise Characteristics for Mass Concrete Using the Physics-Informed Neural Network

**DOI:** 10.3390/ma18204650

**Published:** 2025-10-10

**Authors:** Jae Min Lee, Chang Joon Lee, WoonSeong Jeong

**Affiliations:** Department of Architectural Engineering, Chungbuk National University, 1 Chungdae-ro, Heungdeok-gu, Cheongju 28644, Republic of Korea; woals7654@chungbuk.ac.kr (J.M.L.); cjlee@chungbuk.ac.kr (C.J.L.)

**Keywords:** mass concrete, adiabatic temperature rise characteristics, physics-informed neural network, heat of hydration, parameter identification

## Abstract

This study addresses the inverse problem of identifying adiabatic temperature rise (ATR) characteristics for mass concrete using the Physics-Informed Neural Network (PINN). The characteristics are defined by parameters representing the maximum ATR and temperature increasing rate. The PINN-based identification of these parameters was conducted using virtual experimental data generated through numerical simulation with three different ATR models. To assess the robustness of the PINN in the identification process, noise was introduced into the data. The observation period and noise condition of the data were used as variables to evaluate the performance of PINN-based parameter identification. In addition, 10 independent PINN training sessions were conducted, and the results were statistically analyzed. The identification performance of the unknown parameters was influenced by the observation period. The PINN accurately identified the parameters used in the virtual experiments, even with short-term observation data, regardless of the noise. Statistical analysis indicates that the PINN demonstrates significant reliability and consistency in parameter identification.

## 1. Introduction

The hydration reaction of cement is an exothermic process in which heat is released when cement and water react to form hydrates. A large amount of hydration heat is rapidly released during the hardening process after placement. Concrete structures exposed to the external environment during hydration experience a non-uniform temperature distribution due to the interaction between the generated heat and the surrounding conditions. The temperature difference between the center and the surface of mass concrete caused by the heat of hydration leads to differential volume change, which induces stress within the concrete structure. The thermal stress can cause cracks on the surface or inside the concrete structure, depending on restraints [1]. The cracks can degrade the structural integrity and durability of the concrete structure when additional loads are applied during construction [2,3]. To prevent thermal cracking caused by the heat of hydration and improve the quality of mass concrete structures, the thermal behavior of the concrete mixture to be cast in the field should be understood at the design stage. Based on this result, an appropriate curing method is determined, and temperature management plans are established.

To calculate the temperature distribution inside mass concrete over time through numerical analysis, an ATR model for the concrete mixture to be used during construction is required. The ATR model is determined by fitting a mathematical equation-based model to experimental results obtained from ATR tests. The fitting parameters included in the ATR model mainly represent the final adiabatic temperature rise and temperature increasing rate, which explain the thermal behavior of the concrete mixture. The thermal behavior is affected by several factors, such as initial temperature, cement fineness, supplementary cementitious materials, etc. [1,4,5,6]. For accurate determination of the ATR model, a sophisticated temperature control system is required to maintain adiabatic conditions during testing. Due to the high cost of the test equipment and the difficulty of precise temperature control for the ATR test, the semi-ATR test is sometimes conducted as an alternative.

The semi-ATR test is conducted under weak adiabatic conditions in which heat generated by hydration is partially lost to the surroundings. After the test, the heat loss is compensated to estimate the ATR model. Previous studies have proposed heat compensation techniques based on heat transfer principles. Ng proposed a method to compensate for heat loss using Fourier’s law [7]. The ATR model is estimated as a polynomial equation based on the location and number of temperature measurement points in the semi-ATR test. The method has limitations in that the number of temperature measurement points and the volume of the specimen affect the estimation accuracy. Ahn proposed a method to estimate the ATR model based on Newton’s Cooling Law [8]. The heat loss is compensated for by fitting a solution curve to the temperature at the center point measured in the semi-ATR test, and the ATR model is estimated through this. The fitting period was proposed to be from 72 h after the peak temperature time to the end of the test. While this method has the advantage of estimating the ATR model using a single measurement point, it has limitations such as the ambiguity of defining the test termination time and the impact of the fitting period on estimation accuracy.

The derivative of the ATR curve, determined through the aforementioned methods, is used in numerical analysis to predict the temperature distribution of mass concrete structures under construction and evaluate the possibility of thermal cracking. However, the boundary conditions and constraints used in the numerical analysis for temperature management planning at the design stage may vary depending on the situation. To overcome the limitations arising from discrepancies between real-world conditions and analytical models, data-driven machine learning techniques have been applied in various fields [9,10,11,12]. The approach depends on the quantity and quality of training data. Recently, the PINN has attracted attention as a method for solving inverse problems by integrating data-driven approaches with prior physical knowledge.

The PINN is used for two main purposes: the data-driven solution of PDEs and the data-driven discovery of PDEs [13]. The former involves solving governing equations to obtain solutions like traditional numerical analysis, while the latter is used to estimate unknown parameters included in the governing equations. Unlike conventional data-driven machine learning techniques, the PINN integrates physical constraints such as governing equations, boundary conditions, and initial conditions, enabling reliable analysis in scientific and engineering fields. The PINN demonstrates strong predictive performance with a small amount of data, even in a spatiotemporal range out of the training range [14]. In addition, the PINN has an advantage in terms of computational cost by using a mesh-free strategy, unlike traditional numerical methods. Due to these benefits, the PINN is widely used for solving both forward and inverse problems in various fields. For example, Zhang et al. used the PINN to estimate state variables of structural systems through domain decomposition [15]. Wan et al. applied the PINN to estimate the chloride diffusion coefficient in concrete and predicted chloride distribution in concrete [16]. Zhang et al. demonstrated the superiority of the PINN in analyzing rheological behavior by evaluating the thixotropic properties of cementitious materials [17].

Traditional ATR and semi-ATR tests have inherent limitations. The ATR test is a costly and technically sophisticated process because it requires expensive equipment and precise temperature control to maintain strictly adiabatic conditions. Estimation of the ATR curve through the semi-ATR test is significantly affected by the measurement location and test duration. These issues may reduce the reliability of the identified ATR model in field applications. In contrast, the PINN integrates observation data with physical laws, enabling stable parameter identification and physically consistent solutions even with short-term or noisy datasets. Moreover, it is relatively less sensitive to the observation interval. Such capabilities highlight the potential of the PINN to overcome the shortcomings of conventional methods, offering a more efficient and reliable tool for identifying the hydration heat characteristics for mass concrete.

The objective of this study is to identify the ATR parameters for mass concrete using the PINN. The identification is conducted with virtual experimental data generated through FE simulations. The PINN identifies the ATR parameters with only the data in the virtual experiment data. The performance of the PINN in parameter identification is evaluated by comparing the identified parameters with the values used in the virtual experiments. In addition, noise is added to the virtual experimental data to verify the robustness of the PINN in identifying the unknown parameters.

This paper is organized as follows. Section 2 describes the physical modeling methods for heat transfer analysis and the operational principles of PINNs. Section 3 presents the material parameters used in the virtual experiments and details of the PINN architecture and data preprocessing. Section 4 presents the identification results of the unknown parameters. Finally, Section 5 summarizes the conclusions.

## 2. Physical Modeling and Computational Method

This section introduces the theoretical background necessary for numerically simulating the heat conduction phenomenon in hydrating concrete. It also presents three different types of models that represent the heat generation of concrete. In addition, the operational principles of the PINN used to identify the unknown parameters are briefly explained.

### 2.1. Governing Equation for the Heat Transfer Problem

When water is added to cement, hydration products are formed, and heat of hydration is released. To predict the temperature distribution in concrete over time, a heat generation model should be applied as a heat source. The 1D heat transfer governing equation, which includes the heat source, is defined based on Fourier’s law, as shown in Equation (1).

(1) $$\rho c_{p}\frac{\partial T}{\partial t}=k\frac{\partial^{2}T}{\partial x^{2}}+G(t)$$

where T is temperature, x is position, t is time, ρ is density ($kg/m^{3}$), $c_{p}$ is heat capacity (kcal/kg·°C), k is conductivity (kcal/m·h·°C), and G(t) is the rate of heat generation ($kcal/m^{3}\cdot h$).

The initial condition at *t* = 0 indicates the concrete placement temperature, as shown in Equation (2):

(2) $$T=T_{0},\;\mathrm{for}\;t=0$$

When concrete is exposed to the external environment after placement, the heat released by hydration dissipates from the concrete surface through convection and radiation. For simplicity in analysis, radiation is generally considered together with convection conditions. The convective heat transfer at the concrete surface is expressed as shown in Equation (3):

(3) $${\left.-k\frac{\partial T}{\partial x}\right|}_{s}=h_{c}\left(T_{e}-T_{s}\right)$$

where $h_{c}$ is the convection coefficient, $T_{e}$ is the ambient temperature, and $T_{s}$ is the concrete surface temperature.

### 2.2. Adiabatic Temperature Rise Model

To perform thermal analysis for mass concrete, it is necessary to understand the heat generation characteristics of the concrete mixture to be placed on site. The characteristics, which are expressed as the amount of temperature rise and the temperature increasing rate under adiabatic conditions, are affected by many factors, including the mix proportion, type of cement, chemical composition, and fineness. Due to the complex relationships among the individual factors, it is extremely difficult to estimate the heat generation characteristics of the concrete mixture using simple guidelines. Therefore, the characteristics used in thermal analysis are typically estimated by fitting a formula-based model to the results from ATR tests and determining the unknown parameters. Models defined as functions of time are widely used, and one of the following three formulas is selected for fitting: exponential formula, hyperbolic formula, or complex formula.

- Exponential formula:

(4) $$T\left(t\right)=T_{\infty }\left(1-\exp\left(-rt\right)\right)$$

(5) $$G(t)=\rho c_{p}\frac{dT\left(t\right)}{dt}=\rho c_{p}rT_{\infty }\exp\left(-rt\right)$$

- Hyperbolic formula:

(6) $$T\left(t\right)=T_{\infty }\frac{t}{n+t}$$

(7) $$G\left(t\right)=\rho c_{p}\frac{dT\left(t\right)}{dt}=\rho c_{p}T_{\infty }\left(\frac{1}{n+t}-\frac{t}{{\left(n+t\right)}^{2}}\right)$$

- Complex formula:

(8) $$T\left(t\right)=T_{\infty }\left(1-\exp\left(-at^{b}\right)\right)$$

(9) $$G\left(t\right)=\rho c_{p}\frac{dT\left(t\right)}{dt}=\rho c_{p}T_{\infty }abt^{b-1}\exp\left(-at^{b}\right)$$

where $T\left(t\right)$ is the temperature over time under adiabatic conditions (°C), $G\left(t\right)$ is the rate of heat generation ($kcal/\left(m^{3}h\right)$), $T_{\infty }$ is the final amount of temperature rise (°C), and r, n, a, and b are material constants that parameterize the temperature rising rate. Equations (4), (6) and (8) were proposed by the U.S. Bureau of Reclamation to determine the ATR characteristics of concrete used in dam construction [18,19]. The mathematical forms are widely used not only to represent the heat generation characteristics but also to describe the development of concrete properties, such as the degree of hydration and strength. Equations (5), (7) and (9) are directly used as functions in thermal analysis. The parameters $T_{\infty }$, r, n, a, and b included in these functions are set as unknown parameters when solving the inverse problem using the PINN.

### 2.3. PINN

The PINN is a data-driven machine learning technique integrating physical constraints such as governing equations, initial conditions, and boundary conditions during the training process. This feature provides advantages compared to numerical analysis when solving inverse problems using the PINN. Numerical analysis provides stable and accurate solutions when the governing equations and boundary conditions are explicitly defined. However, if the conditions for numerical analysis do not sufficiently reflect the system, the analysis may yield inaccurate solutions. However, because the PINN is trained by complementing global responses of the system and physical constraints, it can generalize the system’s behavior even when using unknown conditions or a small amount of data. Therefore, the PINN can be used as a powerful and effective tool for solving inverse problems by combining the global response of the system and physical constraints.

To solve inverse problems related to heat transfer using the PINN, a fully-connected neural network (FCNN) is required to derive the solution of the governing equation. The FCNN receives position and time as inputs and predicts the temperature, as shown in Equation (10). The predicted temperature is an approximate solution to the governing equation and is used to compute the loss functions. The residual of the governing equation, Equation (11), is derived from the difference between its left-hand side and right-hand side from Equation (1), representing a measure of how well the predictions satisfy the physical law. The PINN is trained to minimize the residual, and the predicted temperature gradually converges to the analytical solution.

(10) $$\bar{T}=NN\left(x,t\right)$$

(11) $$f(x,t,\gamma )=\rho c_{p}{\bar{T}}_{t}-k{\bar{T}}_{xx}-G\left(t\right)$$

where $\bar{T}$ is the temperature predicted by the FCNN, $f\left(x,t,\gamma \right)$ is the residual of the governing equation, ${\bar{T}}_{t}$ is a derivative of temperature with respect to time, ${\bar{T}}_{xx}$ is the second derivative of temperature with respect to position, and γ is unknown parameters included in G(t).

The total loss function for training the PINN consists of a weighted sum of the loss functions for the governing equation, initial conditions, boundary conditions, and observation data, as shown in Equation (12):

(12) $$Loss=W_{Data}Loss_{Data}+W_{PDE}Loss_{PDE}+W_{IC}Loss_{IC}+W_{BC}Loss_{BC}$$

where $Loss_{Data}$ is the loss function corresponding to observation data, $Loss_{PDE}$ is the loss function corresponding to the governing equation, $Loss_{IC}$ is the loss function corresponding to the initial condition, $Loss_{BC}$ is the loss function corresponding to the boundary condition, and $W_{Data}$, $W_{PDE}$, $W_{IC}$, and $W_{BC}$ are weights corresponding to each loss function. Each loss function is defined as the mean square error as in Equations (13)–(16):

(13) $$Loss_{PDE}=\frac{1}{N_{PDE}}\sum_{i=1}^{N_{PDE}}f{\left(x,t,\gamma \right)}^{2}$$

(14) $$Loss_{Data}=\frac{1}{N_{Data}}\sum_{i=1}^{N_{Data}}{\left(T_{Data}-\bar{T_{i}}\right)}^{2}$$

(15) $$Loss_{IC}=\frac{1}{N_{IC}}\sum_{i=1}^{N_{IC}}{\left(T_{IC}-\bar{T_{i}}\right)}^{2}$$

(16) $$Loss_{BC}=\frac{1}{N_{BC}}\sum_{i=1}^{N_{BC}}{\left(T_{BC}-\bar{T_{i}}\right)}^{2}$$

where $N_{PDE}$ is the number of collocation points, $N_{Data}$ is the number of observation data, $N_{IC}$ is the number of initial conditions, $N_{BC}$ is the number of boundary conditions, and $T_{Data}$, $T_{IC}$, and $T_{BC}$ are observation data, initial temperature, and boundary temperature, respectively.

The total loss function is minimized by the optimizer during the training process, as shown in Equation (17). This process is repeated until the loss becomes smaller than a pre-defined threshold or the maximum number of iterations is reached. During training, the unknown parameters included in G(t) are treated as learnable parameters and converge to the optimal values simultaneously with the adjustment of network parameters.

(17) $$\left\{\bar{\theta },\bar{\gamma }\right\}=\underset{\left\{\theta ,\gamma \right\}}{argmin}\;Loss\left(\theta ,\gamma \right)$$

where $\bar{\theta }$ is the estimated parameters of the FCNN, $\bar{\gamma }$ is the estimated unknown parameters included in G(t), and θ is the parameters of the FCNN used for prediction.

A key concept for implementing the PINN is automatic differentiation. Derivatives of temperature with respect to time or position are required to compute the loss function corresponding to the governing equation. Automatic differentiation leads to directly computing the derivatives of the network output with respect to its inputs using the chain rule. When traditional numerical differentiation is used, the analysis results may vary depending on the discrete interval or the order of the difference. However, automatic differentiation is based on symbolic rules within the computation graph, allowing it to avoid discretization or truncation errors [20].

## 3. Virtual Experiment and PINN Implementation

**Figure 1:**
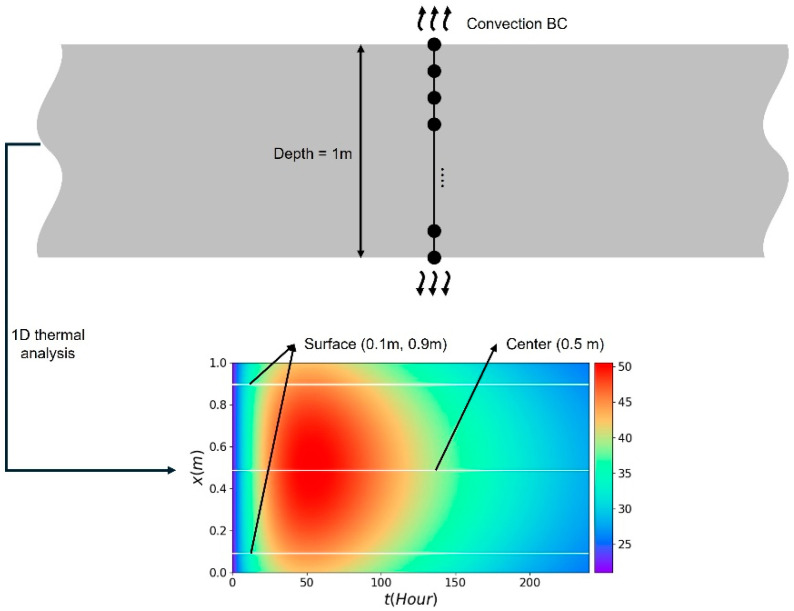
Schematic diagram of the virtual experiment.

In this section, settings for the virtual experiment and PINN implementation are introduced. Figure 1 illustrates the schematic diagram of the virtual experiment to generate temperature data at the surface and center for the identification of the ATR parameters. The virtual experiment data were obtained through 1D thermal analysis, and the material properties used were referenced from previous research.

### 3.1. Virtual Experiment

The virtual experiment was conducted to generate the temperature distribution with time in mass concrete. FE simulation for data generation has been used to simulate the thermal behavior of cast-in-place mass concrete members and shows good agreement [21,22]. The virtual experiment consists of three steps: creating a 1D geometric model, discretizing the spatiotemporal domain, as shown in Figure 1. A 1 m geometry was discretized into 100 elements in the spatial domain. The analysis duration was set to 240 h and discretized into 0.1 h intervals in the temporal domain. Convection boundary conditions were applied at both ends, allowing heat generated by hydration to dissipate. The ambient temperature $T_{e}$ was set to 20 °C, and the initial temperature was set to 21 °C. The material properties, such as specific heat, thermal conductivity, density, and convection coefficient, used in the virtual experiment are referenced from previous research and summarized in Table 1 [23,24,25].

**Figure 2:**
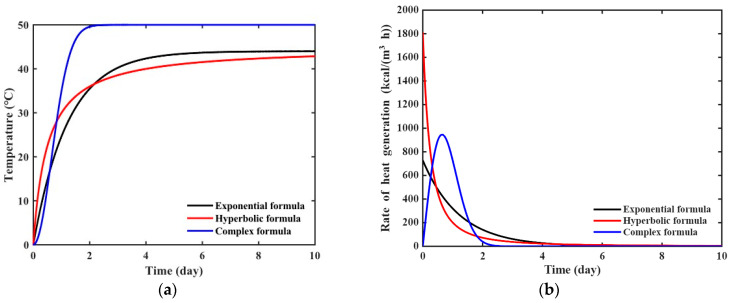
Heat generation curve of virtual materials: (**a**) ATR curve; (**b**) rate of heat generation.

The ATR parameters of virtual materials based on three different equations were assumed as shown in Table 2. The ATR curve and the rate of heat generation for each virtual material are presented in Figure 2. Figure 2a,b illustrate the ATR curves and the rate of heat generation, respectively, obtained by substituting the parameters in Table 2 into Equations (4), (6) and (8) for ATR curves and Equations (5), (7) and (9) for the rate of heat generation. The rate of heat generation in Figure 2b was used to obtain virtual experimental data through numerical analysis.

**Figure 3:**
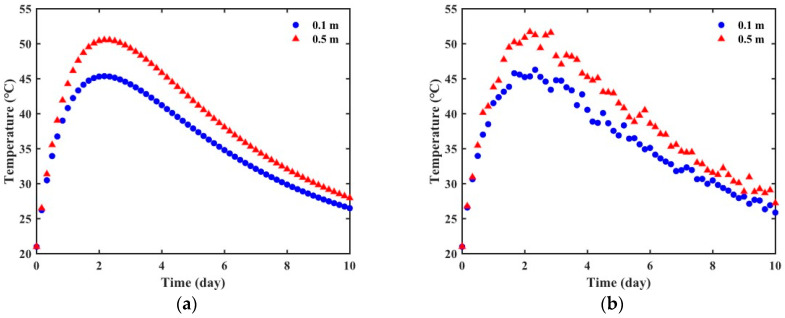
Virtual experimental data: (**a**) noise-free data; (**b**) noisy data.

The solution of the governing equation obtained through FE simulation is mathematically ideal. However, the data measured through actual experiments may differ from the analysis results due to noise caused by uncontrollable factors such as measurement errors and inhomogeneity of concrete materials. To verify the robustness of the PINN in the identification process, noise was added to the data obtained from FE simulation using Equation (18). The noisy data were then used as virtual experimental data.

(18) $$T_{n}=T+\delta_{0}\omega T$$

where $T_{n}$ is the noisy temperature data, T is the temperature data from the FE simulation, $\delta_{0}$ is the corruption level of noise, and ω is a random variable following the standard normal distribution. Figure 3 shows the virtual experimental data generated based on the exponential formula using the parameters in Table 2. A noise level of 2% was introduced into the FE simulation data. The accuracy of temperature measurements obtained from thermocouples or resistance temperature detectors (RTDs) is typically within ±0.5 °C to ±2 °C. Considering the temperature rise in the mass concrete structures exceeding 50 °C, the level can be regarded as a reasonable approximation observed in a laboratory or field environment.

### 3.2. PINN Architecture

**Figure 4:**
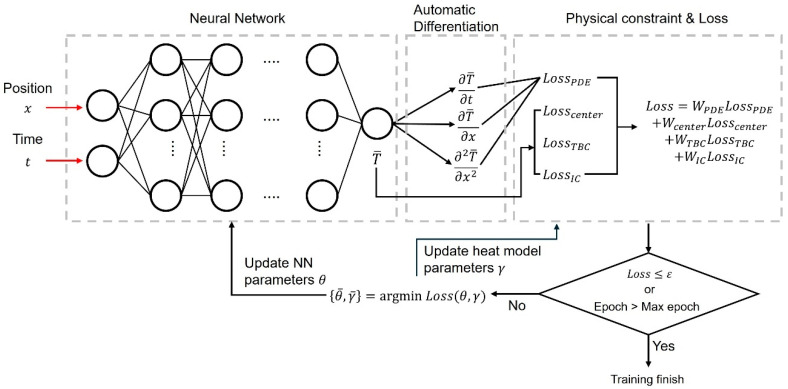
Schematic diagram of the PINN training process.

The training process for the identification of unknown parameters using virtual experimental data is illustrated in Figure 4. The input and output variables of the FCNN vary depending on the governing equation. In this study, the input and output variables were defined based on the 1D transient heat transfer equation. The input variables consist of spatial positions along a single coordinate axis and time, while the output variable is the temperature at the given position and time. The nonlinear relationship between input and output variables is mapped through weighted linear combinations of FCNN parameters and a nonlinear transformation by activation functions in the hidden layer. The hidden layers play a crucial role in representing the system’s nonlinearity, and their size influences the generalization performance. If the hidden layers are too large, the model complexity increases, potentially leading to slower training and overfitting. Conversely, if they are too small, the model may fail to capture the complexity of the data. Since there are no definitive guidelines for determining the appropriate hidden layer size, it depends on user experience. In this study, the FCNN with 4 hidden layers and 20 neurons in each layer was constructed. Tanh was used as the activation function.

The Xavier initialization method was used to initialize the weights and biases before training. The purpose of the method is to initialize the weights so that the variance of the input data is equal to the variance of the output data. The method enhances convergence stability during training, particularly when Tanh is used as an activation function [26].

To ensure stable training of the PINN, hyperparameters must be determined. The hyperparameters include the learning rate, optimizer, and maximum number of epochs, which were selected through a trial-and-error process. The Adam optimizer with a learning rate of 0.001 was used for minimizing the total loss function. The maximum number of epochs was set to 200,000. PINN was implemented using the PyTorch 2.2 framework to identify the ATR parameters for mass concrete.

### 3.3. Data Setting for Training

The spatial domain of the data obtained from the virtual experiment is $\left[x_{min},x_{max}\right]=\left[0,\;1\;m\right]$, and the temporal domain is $\left[0,t_{max}\right]=\left[0,\;240\;h\right]$. Convection boundary conditions were applied at both ends of the geometry in the virtual experiment. The convection coefficient, which determines the amount of heat dissipation to the ambient air, is influenced by factors such as the surface roughness of concrete and wind speed. In field situations where many variables exist, it is difficult to accurately determine the convection coefficient by taking these factors into account. To overcome this limitation, this study used the temperature observed near the surface when constructing the loss function corresponding to the boundary conditions. The observed temperature is a global response derived from the complex interactions between heat generation in concrete and heat dissipation to the air. The temperature boundary conditions alleviate concerns about considering complex external boundary conditions.

**Figure 5:**
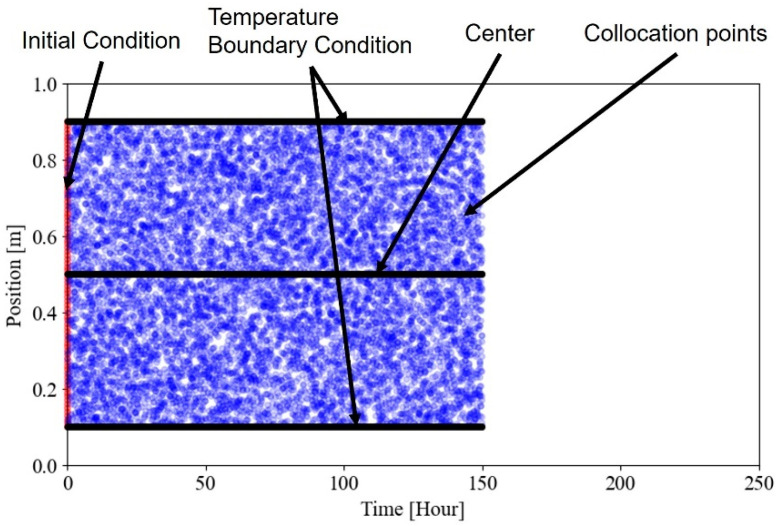
Example of a domain used for the loss function.

Figure 5 illustrates an example of the points used for constructing the loss function corresponding to observation data and physical constraints. Temperature measurements were taken at three locations within the 1 m concrete, 0.1 m, 0.5 m, and 0.9 m from the bottom (0.0 m), and these were used as observation data. The temperatures at 0.1 m and 0.9 m were specifically applied as data for the upper and lower temperature boundary conditions, respectively. The total 240 h data were divided into subsets of 12 h, 24 h, 48 h, 72 h, and 168 h, which were then used as observed data for training the PINN. The performance of PINN-based parameter identification was evaluated with respect to the observation period. The initial condition data consisted of 80 temperature values within the range of $\left[0.1\;m,0.9\;m\right]$. A total of 10,000 collocation points were randomly selected within the spatial domain of $\left[0.1\;m,0.9\;m\right]$ and the temporal domain of the observation period.

The unknown parameters are initialized as random values within the range of 0 to 1. Since the randomly initialized unknown parameters differ for each training session, they lead to different parameter identification results. To evaluate the consistency of the PINN under varying initial values, the mean absolute percentage error (MAPE) between the identified parameters from 10 independent training sessions and the target values was calculated using Equation (19). The target values are the parameters used in the virtual experiment, as presented in Table 2.

(19) $$MAPE=\frac{1}{N}\sum_{i=1}^{N}\left|\frac{\gamma_{i}-{\bar{\gamma }}_{i}}{\gamma_{i}}\right|\times 100$$

where N is the number of independent training sessions, $\gamma_{i}$ is the target value, and ${\bar{\gamma }}_{i}$ is the identified parameter.

## 4. Results and Discussion

### 4.1. Results

**Figure 6:**
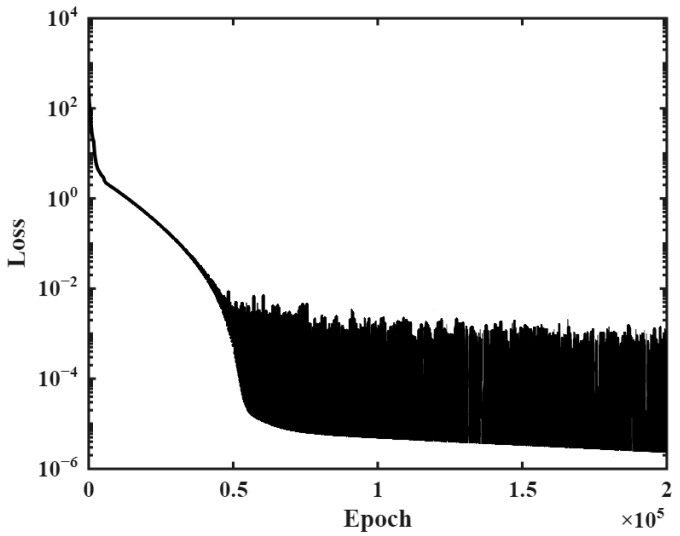
Total loss decrease with training (noise-free data).

**Figure 7:**
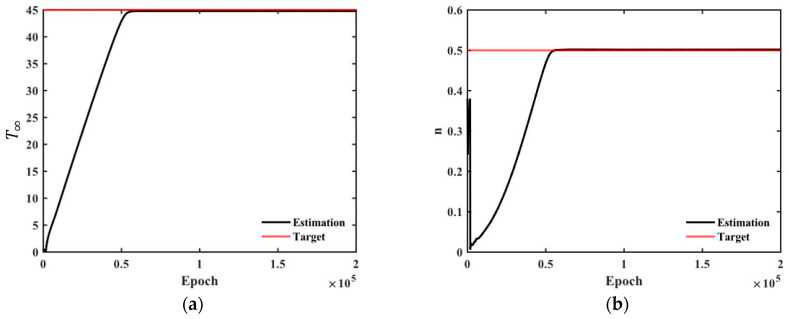
Identification process of the unknown parameters included in the hyperbolic formula: (**a**) $T_{\infty }$; (**b**) n.

The PINN minimizes not only the residuals of the observed data but also the residuals associated with physical constraints during training. Figure 6 illustrates the decrease in the total loss function with the training process. As the number of epochs increases, the loss function continuously decreases. The PINN identifies the ATR parameters during training. Figure 7 shows the identification process of the unknown parameters using noise-free data based on the hyperbolic formula. The initially randomized unknown parameters gradually converge toward the target values during the training process.

**Figure 8:**
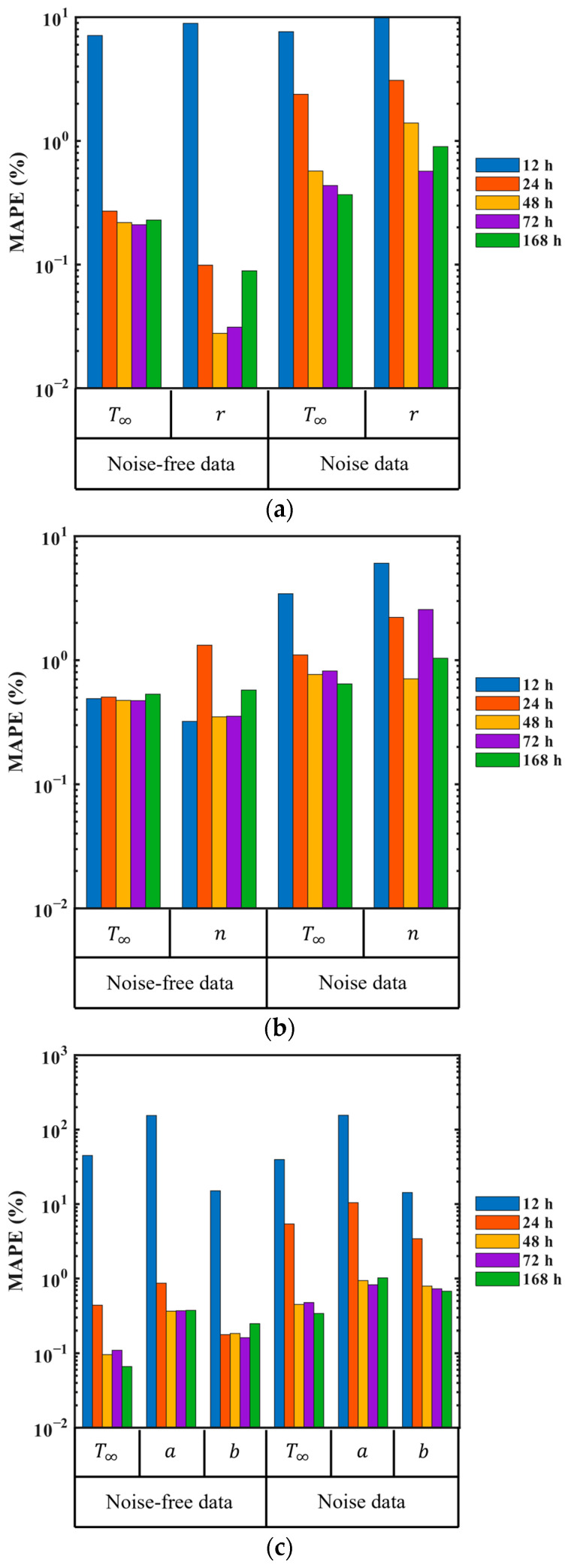
MAPE of the identified unknown parameters for each formula: (**a**) exponential formula; (**b**) hyperbolic formula; (**c**) complex formula.

Figure 8 presents the MAPE of PINN-based parameter identification results. This metric represents the average relative error between the identified unknown parameters and the target values through 10 independent training sessions using the same dataset. Since the unknown parameters are randomly initialized in each training session, the results help assess the PINN’s capability in identifying these parameters. The observation period plays a crucial role in accurately identifying the unknown parameters. When using noise-free observation data from 24 h, 48 h, 72 h, and 168 h periods, the error remains within 1%. However, when using noise-free 12 h observation data, the relative error exceeds 7%, indicating that 12 h of observed data is insufficient for accurately identifying the unknown parameters. On the other hand, when using noisy data, even a 24 h observation period results in a significantly larger error. The results indicate that the observation period should be adjusted based on the presence of noise in the data.

The PINN has robustness against noise in identifying the unknown parameters. In Figure 8, the identification results using noisy data show a greater number of errors compared to those using noise-free data. This indicates that the presence of noise introduces some variability into the parameter identification process. However, despite the increase in MAPE, noise does not significantly degrade the overall identification performance. When the parameters are identified using observation data longer than 48 h, the identification error remains within 1%.

Table 3 and Table 4 present the statistical analysis of the identified unknown parameters based on the mean and standard deviation through 10 independent training sessions, considering the presence or absence of noise. The results provide valuable insights into the consistency of PINN-based identification by quantitatively comparing the effects of observation period and noise on the parameter identification process. Close approximation to target values was achieved when noise-free data with at least a 24 h observation period and noisy data with at least a 48 h observation period were used for identification. Furthermore, regardless of the observation period and noise, most standard deviations remain below 1, indicating minimal variability. These findings demonstrate that PINN-based identification can accurately capture the ATR parameters for mass concrete from observation data while maintaining reliability and consistency.

### 4.2. Discussion

The ATR parameters of concrete were identified using virtual experimental data generated through numerical analysis. The inverse problem-solving performance based on PINN was evaluated over the observation period using three points: one at the center and two at the surface. In most cases, the parameters initialized with random values converged to the target values as training progressed, as shown in Figure 7. The unknown parameters of virtual materials based on the exponential formula and hyperbolic formula approximate the target values even when using 12 h observation data. In contrast, the unknown parameters of virtual materials based on the complex formula exhibited relatively larger errors even with 12 h of observation data. The result can be attributed to the loss function corresponding to the governing equation used during PINN training.

In Equation (13), when constructing the loss function corresponding to the governing equation, the rate of heat generation formula from Equations (5), (7) and (9) is used as a heat source. As shown in Figure 2b, the rate of heat generation by the exponential formula and the hyperbolic formula reaches its maximum at *t* = 0 and continuously decreases over time. However, the rate of heat generation by the complex formula reaches its peak at *t* = 15.6 h and decreases thereafter. In the rate of heat generation function, density and specific heat are known values, while the final amount of temperature rise $T_{\infty }$ is the dominant parameter that determines the maximum rate of heat generation. The parameters r,n,a, and b are used to express the shape of the rate functions. The features of the rate function are directly reflected in the temperature data generated through numerical analysis. The inclusion of the maximum rate values within the observation period affects the performance of PINN-based ATR parameter identification.

**Figure 9:**
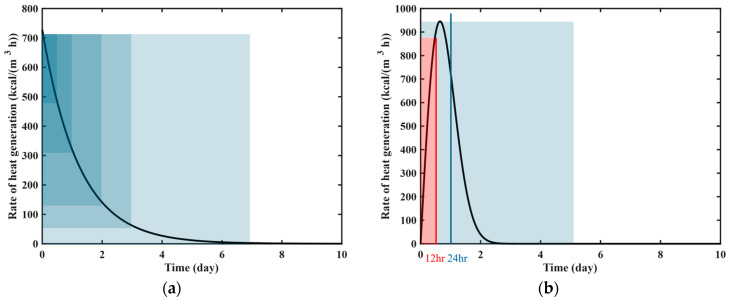
The rate of heat generation function: (**a**) exponential formula; (**b**) complex formula.

Figure 9 illustrates the rate of heat generation functions. Figure 9a represents the rate function of the exponential formula, which is similar to that of the hyperbolic formula. The shaded areas in Figure 9a indicate observation periods of 12 h, 24 h, 48 h, 72 h, and 168 h from left to right. The maximum rate is observed at *t* = 0 and decreases monotonically with time throughout the whole duration. This heat generation behavior is inherently embedded in the data regardless of the observation period. The temperature information in the observation data is incorporated not only in the loss function corresponding to the data but also in the loss function corresponding to the governing equation. The PINN is trained to fit the data while satisfying the physical constraints imposed by the governing equation. Based on the mathematical formula included in the governing equation, the unknown parameters progressively approximate the target values during training. Therefore, when using virtual experimental data based on the exponential formula and hyperbolic formula, PINN can identify the parameters by analyzing the temperature rise in the observation data, even with short-term training data.

Figure 9b indicates the rate function of the complex formula, which increases before *t* = 15.6 h and then decreases. The PINN can identify the unknown parameters based on whether the maximum temperature is included in the observation period. The inclusion of the maximum rate depends on the observation period. In Figure 9b, the red-shaded area represents the 12 h observation period, while the blue-shaded area corresponds to observation periods that extend beyond the peak heat generation time. The 12 h observation data do not include the peak rate, whereas the 24 h observation data capture this peak. Therefore, a 12 h observation period is insufficient for accurately identifying the parameters included in the complex formula.

**Figure 10:**
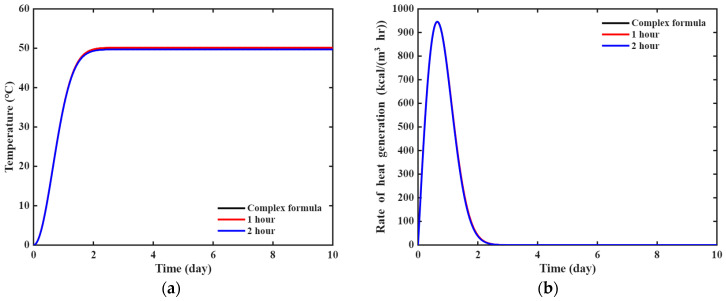
Effect of the observation interval on the heat generation model: (**a**) temperature rise; (**b**) rate of heat generation.

A parametric study was conducted with respect to the observation interval. The observation data were sampled at 1 h and 2 h intervals within a 24 h period. Figure 10 illustrates the temperature rise and heat generation rate of the complex formula plotted over the identified parameters based on the sampled data. The parameters ($T_{\infty },\;a,\;b$) identified by the PINN were (50.1624, 1.1968, 1.9901) for the 1 h interval case and (49.6920, 1.2131, 2.0029) for the 2 h interval case. The quantities from both cases were approximated with the actual complex formula, and no significant differences were exhibited. This indicates that the PINN framework is robust in identifying the unknown parameters.

As a result, it was confirmed that the ATR parameters could be accurately identified using the PINN. The heat generation behavior of cement hydration in concrete exhibits a pattern similar to that of the complex formula function. Factors such as the water-to-cement ratio, unit cement content, cement fineness, and chemical admixtures influence the hydration process of concrete. This implies that when applying PINN-based parameter identification to actual experimental data, different observation periods should be considered accordingly.

## 5. Conclusions

This study has demonstrated that the PINN can accurately identify ATR parameters for mass concrete using virtual experimental data with high reliability. Virtual experimental data were generated through 1D heat transfer simulations based on three types of ATR models. The PINN not only fits the observation data but also trains to satisfy physical constraints such as governing equations, initial conditions, and boundary conditions and simultaneously identifies the unknown parameters embedded in the ATR model. Temperature data observed near the surface and at the center were used as training data for the PINN, and the unknown parameters were randomly initialized before training. The performance of the PINN in identifying the unknown parameters was evaluated based on the presence of noise in the data and different observation periods. It was shown that the PINN can achieve accurate identification even with short-term observation data. In addition, robustness was verified through noisy data. The randomly initialized unknown parameters converged to the target values during training. It can be seen that through 10 independent training sessions and statistical analysis, the PINN can identify the ATR parameters with high consistency and reliability.

The identification of ATR parameters for mass concrete using the PINN offers significant advantages compared to the heat loss compensation method in terms of observation period and accuracy. The significant reduction in required observation time can lead to more efficient quality control and earlier decision-making for mass concrete construction projects. In addition, accurate identification capacity with noisy data can help improve the structural integrity of mass concrete structures through real-time training using field-measured data.

## Figures and Tables

**Table 1:** Material properties used in the virtual experiment.

	k (kcal/m °C h)	$c_{p}$ (kcal/kg °C)	ρ (kg/m^3^)	$h_{c}$ (kcal/m^2^ °C h)
Value	1.81	0.21	2300	2.67

**Table 2:** Parameter setting of the ATR curve for virtual experiment.

	$T_{\infty }\left({}^{\circ}C\right)$	r	n	a	b
Exponential formula	44	0.822	-	-	-
Hyperbolic formula	45	-	0.5	-	-
Complex formula	50	-	-	1.2	2

**Table 3:** Statistical analysis of the identified unknown parameters using noise-free data.

			12 h	24 h	48 h	72 h	168 h
Exponential formula	$T_{\infty }$	Mean	40.8618	43.8810	43.9037	43.9076	43.8992
		Std	0.4204	0.0032	0.0001	0.0022	0.0117
	r	Mean	0.8954	0.8228	0.8222	0.8219	0.8213
		Std	0.0111	9.59 × 10^−5^	2.79 × 10^−5^	0.0006	0.0008
Hyperbolic formula	$T_{\infty }$	Mean	44.7795	44.7730	44.7864	44.7874	44.7605
		Std	0.0015	0.0316	0.0016	0.0024	0.0347
	n	Mean	0.5016	0.5066	0.5017	0.5017	0.5028
		Std	2.76 × 10^−5^	0.0109	3.51 × 10^−5^	8.28 × 10^−5^	0.0020
Complex formula	$T_{\infty }$	Mean	27.5522	49.7811	49.9520	49.9451	49.9668
		Std	3.2680	0.1392	0.0139	0.0123	0.0080
	a	Mean	3.0566	1.2103	1.2043	1.2044	1.2044
		Std	0.5093	0.0048	8.32 × 10^−5^	8.69 × 10^−5^	0.0002
	b	Mean	2.3008	2.0011	1.9963	1.9967	1.9950
		Std	0.0951	0.0041	0.0008	0.0008	0.0006

**Table 4:** Statistical analysis of the identified unknown parameters using noisy data.

			12 h	24 h	48 h	72 h	168 h
Exponential formula	$T_{\infty }$	Mean	42.2256	44.3003	43.9780	43.9045	43.9085
		Std	4.1866	1.3205	0.2826	0.2199	0.1745
	r	Mean	0.8767	0.8153	0.8189	0.8203	0.8200
		Std	0.1125	0.0333	0.0124	0.0062	0.0105
Hyperbolic formula	$T_{\infty }$	Mean	45.5134	45.0134	44.6723	44.6314	44.7273
		Std	1.8914	0.6848	0.2692	0.1759	0.2785
	n	Mean	0.5153	0.5074	0.4999	0.5076	0.4995
		Std	0.0347	0.0121	0.0046	0.0207	0.0062
Complex formula	$T_{\infty }$	Mean	30.1829	48.0259	49.8273	49.9782	49.8641
		Std	7.4667	2.7617	0.2339	0.3297	0.1609
	a	Mean	3.0679	1.3007	1.2072	1.2046	1.2071
		Std	1.5318	0.1238	0.0110	0.0120	0.0119
	b	Mean	2.2858	2.0556	1.9932	1.9939	1.9999
		Std	0.1744	0.0685	0.0187	0.0161	0.0178

## Data Availability

The original contributions presented in this study are included in the article; further inquiries can be directed to the corresponding author.

## Abbreviations

The following abbreviations are used in this manuscript:

ATR	Adiabatic Temperature Rise
PINN	Physics-Informed Neural Network
FCNN	Fully-Connected Neural Network
MAPE	Mean Absolute Percentage Error
